# *RET*融合型非小细胞肺癌的研究进展

**DOI:** 10.3779/j.issn.1009-3419.2021.102.22

**Published:** 2021-08-20

**Authors:** 玉军 郑, 巍 姜, 东妍 陈, 颜君 李, 璐璐 代, 磊 黄, 明吉 王

**Affiliations:** 116001 大连，大连市友谊医院肿瘤内科 Department of Oncology, The Friendship Hospital of Dalian, Dalian 116001, China

**Keywords:** 肺肿瘤, 转染原癌基因, 融合基因, Lung neoplasms, Transfection proto-oncogene gene, Fusion gene

## Abstract

过去的20年来，随着分子生物学研究的不断进展，非小细胞肺癌(non-small cell lung cancer, NSCLC)的治疗方法不断发展，靶向治疗使驱动基因突变阳性患者的生存期得到了改善。越来越多的靶点逐渐被发现，针对不同驱动基因的药物将NSCLC的治疗带入了一个前景广阔的靶向时代。在NSCLC的诸多驱动基因中，转染原癌基因(transfection proto-oncogene gene, RET)融合是除了表皮生长因子受体(epidermal growth factor receptor, EGFR)、间变淋巴瘤激酶(anaplastic lymphoma kinase, ALK)及c-ros原癌基因1-受体酪氨酸激酶(c-ros oncogene 1, receptor tyrosine kinase, ROS1)以外又一个重要的新兴靶点，针对RET基因融合的靶向药物不断推陈出新，近来新型高选择性RET抑制剂BLU-667和LOXO-292获得了重要突破。本文将对NSCLC中RET基因融合突变的概述、检测方法、临床病理特征、靶向治疗及耐药后的研究进展进行综述。

## Abstract (en)

In the past 20 years, with the development of molecular biology, the treatment of non-small cell lung cancer (NSCLC) has been developing. Targeted therapy has improved the survival period of patients with positive mutation of tumor driver gene. More and more targets have been found gradually. Drugs targeting different driving genes have brought the treatment of NSCLC into a promising target era. Among the many driving genes of NSCLC, the fusion of transfection proto oncogene (*RET*) is the addition of the epidermal growth factor receptor (*EGFR*), analytic lymphama kinase (*ALK*) and *c-ros* oncogene 1-receptor tyrosine kinase (*ROS1*) are emerging targets. Targeted drugs for *RET* gene fusion have been constantly updated. Recently, new high selective RET inhibitors blu-667 and loxo-292 have made important breakthroughs. This paper will review the review of the fusion mutation of *RET* gene in NSCLC, the detection methods, clinicopathological characteristics, targeted treatment and the research progress after drug resistance.

恶性肿瘤已经成为严重威胁全球人类健康的公共卫生问题之一，2020年最新的统计数据^[1]^显示，肺癌位居全球恶性肿瘤发病第二位，年新发病例约为220.6万例; 死亡首位，每年因肺癌死亡人数约为179.6万例。20世纪末开始，随着分子生物学的迅猛发展，尽管仍有40%非小细胞肺癌(non-small cell lung cancer, NSCLC)的分子发病机制未明，但基础研究陆续发现了肺癌不同类型的驱动基因，由此推动了临床针对NSCLC各种不同驱动基因的个体化精准靶向治疗。针对表皮生长因子受体(epidermal growth factor receptor, *EGFR*)、间变淋巴瘤激酶(anaplastic lymphoma kinase, *ALK*)及*c-ros*原癌基因1-受体酪氨酸激酶(*c-ros* oncogene 1, receptor tyrosine kinase, *ROS1*)等基因变异，靶向药物(如吉非替尼^[2]^、厄洛替尼^[3]^、克唑替尼^[4]^等)针对性强、有效率高，极大地改善了患者的预后。文献^[5-7]^报道染色体重排在NSCLC中的发生率较低(1%-7%)，但靶向治疗有效率高且耐受性好，融合基因变异也因此成为当前肺癌分子靶向治疗研究的热点。转染原癌基因(transfection proto-oncogene gene, *RET*)融合基因在NSCLC患者中，是继*EGFR*和*ALK*等基因之后发现的新型驱动基因，研究^[8]^表明*RET*融合可能是*EGFR*、*ALK*及*ROS1*均未突变肺癌患者的主要发病途径，抑或是此类NSCLC患者致病的新靶点。含有该基因变异的患者具有独特的临床特征，提示该靶点是NSCLC中特异性较高的分子标志物。

## 1 RET融合基因概述

### 1.1 RET的结构及生理功能

*RET*原癌基因是正常发育过程中所需的一种受体酪氨酸激酶(receptor tyrosine kinase, RTK)，1985年最先在转化小鼠的NIH/3T3细胞中被发现^[9]^，由位于染色体10q11.2上的*RET*(在转染过程中重新排列)原癌基因编码，含有21个外显子，全长60 kb，编码一种跨膜酪氨酸蛋白激酶受体，同其他酪氨酸蛋白激酶一样，RET蛋白也是由胞外区、跨膜区和胞内酪氨酸蛋白激酶功能区组成(该区通过细胞外信号转导来调节细胞的分化和增殖)，其中胞外区与细胞间的信号转导有关^[10]^。与其他受体酪氨酸激酶不同，RET不直接与其配体结合，其配体为胶质细胞源性神经营养因子(glial-cell derived neurotrophic factor, GDNF)家族，包括GDNF、NINneuturin、ARTartimin和PSP persephin四种。这些GDNF家族配体(GFL)与GDNF家族受体-α(GFRα)(共受体)结合。随后，GFL-GFRα复合物介导RET同源二聚化，导致RET胞内结构域内酪氨酸残基的反式自磷酸化，激活细胞增殖的信号转导级联途径，包括MAPK、PI3K、JAK-STAT、PKA和PKC路径，从而调节细胞的正常生理功能^[11]^。在胚胎发育过程中，这种激酶在肾脏和肠神经系统的发育中具有重要作用。另外，RET维持组织的稳态也非常重要，包括神经、神经内分泌、造血和男性生殖细胞组织; 因此，RET失活会影响多个系统功能，包括泌尿生殖系统、胃肠系统、呼吸系统和造血系统^[12]^。

### 1.2 RET的致瘤作用

RET结构和功能的变异可导致肿瘤发生，*RET*原癌基因已经被证实与多种恶性肿瘤的发生发展具有相关性，最主要的致癌变异为基因融合和突变。首先是染色体重排^[13-16]^，*RET*基因通过本身断裂再与其他基因接合的方式发生重组，成为一个新的融合基因，产生包含RET激酶结构域和伙伴蛋白二聚化结构域的融合蛋白; 在NSCLC中，至今已经发现了至少12个融合*RET*伴侣基因(*KIF5B-RET*, *CCDC6-RET*, *NCOA4-RET*, *MYO5C-RET*, *EPHA5-RET*, *TRIM33-RET*, *CLIP1-RET*, *ERC1-RET*, *PICALM-RET*, *FRMD4A-RET*, *RUFY2-RET*, *TRIM24-RET RET*)。其中*KIF5B-RET*融合最为常见。对于所有*RET*融合变体，染色体融合导致伴侣基因的卷曲螺旋结构域和RET细胞内激酶结构域之间的混合，尽管有断点，其功能保持不变。*RET*伴侣基因的卷曲螺旋结构域诱导RET不依赖配体的同源二聚化并通过自动磷酸化激活RET酪氨酸激酶结构域。RET融合蛋白的激活机制类似于NSCLC中ALK融合的致癌激活，但与ROS1明显不同。在*EML4-ALK*融合基因中，EML4中的卷曲螺旋结构域与ALK激酶结构域融合，赋予寡聚化和组成型激酶活化，而卷曲螺旋结构域在NSCLC中的*ROS1*融合基因中并不一致地存在，并且它们不必驱动肿瘤发生^[14]^。*RET*的第二常见致癌变异为直接或间接突变^[17-19]^。*RET*激活突变可导致细胞外半胱氨酸残基替代氨基酸，破坏分子内二硫键，形成新的分子间共价二硫键，导致配体无关的二聚化; 这些体细胞或种系的改变与人类某些癌症的发病机制相关。激活的*RET*重排和突变具有相同的致癌基因特征，即均通过形成二聚体使RET活化，从而确定了相同的治疗靶点。例如，不同融合伴侣的RET融合蛋白和各种位点*RET*突变蛋白在体外和体内转染原代细胞，并以不依赖配体的方式激活下游信号通路。RET除融合和突变外的其他机制也可能参与RET介导的肿瘤发生。首先，在没有明确的*RET*基因改变的情况下，RET表达的增加可能有助于某些肿瘤的生长和生存。RET已被证明^[20, 21]^是雌激素受体(estrogen receptor, ER)的直接转录靶点，这一发现与胚系ESR2(编码ERβ，抑制RET活化)失活突变的罕见家族性甲状腺髓样癌(medullary thyroid cancers, MTC)中ER介导的RET表达增加可能一致^[22]^。另有研究^[23-25]^表明，ER阳性乳腺癌内分泌治疗获得性耐药时，检测RET表达增加，通过对RET的抑制可以恢复乳腺癌内分泌治疗的敏感性。第二，RET已被确定为多种肿瘤细胞系中主要组织相容性复合体(major histocompatibility complex, MHC) I类表达的强负调节因子^[26]^。这一发现提示RET抑制可能上调抗癌免疫应答。

临床上，*RET*基因的致瘤作用最初发现于乳头状甲状腺癌(papillary thyroid carcinomas, PTCs)，不同形式的染色体异位和插入导致*PTCs/RET*融合基因的形成，被认为是乳头状甲状腺癌的驱动突变(10%-20%)^[27]^;在MTC、NSCLC、多发性内分泌腺瘤2型(multiple endocrine neoplasia type 2, MEN2)、先天性巨结肠等恶性肿瘤中，均发现存在*RET*基因融合现象。NSCLC常见的*KIF5B-RET*型融合突变，*KIF5B*和*RET*分别位于10p11.22和10q11.21，相距10.6 Mb，通过臂间倒位后相融合，形成*KIF5B-RET*融合基因，由KIF5B的N端残基和RET激酶的C端残基组成。KIF5B和RET双螺旋DNA的融合断点并非完全一致，目前已发现KIF5B-RET至少存在7种变体。其中K15:R12是最常见的一种变体，占*KIF5B-RET*融合基因变体的60%-70%。正常肺组织中无KIF5B-RET及RET的表达，但在有RET融合基因的肺腺癌组织中表达水平较高，而且这种融合基因从未在其他类型的腺癌中发现，包括卵巢癌和结肠癌^[28]^。RET的另一种常见致癌变异为*RET*基因突变，研究^[29]^发现，体细胞*RET*突变见于约65%的散发性MTC中，而这些MTC又占所有MTC的3/4。多数为*RET* M918T突变，其他*RET*突变，包括E768D和A883F44;除了点突变，还检测到小的插入和缺失。与*RET*重排一样，随着越来越多地使用下一代测序(next-generation sequencing, NGS)平台来检测人类肿瘤标本，发现了多个瘤种中经典的和新的*RET*突变包括乳腺癌中的*RET* C634R，子宫内膜癌和默克尔细胞癌中的*RET* E511K，副神经节瘤和非典型类肺癌中的*RET* M918T，结直肠癌、脑膜瘤、胃肠道间质瘤(gastrointestinal stromal tumors, GIST)和肝癌中的*RET* V804M突变等^[30]^。

## 2 RET的检测方法

精准检测是精准治疗的前提。*RET*基因融合在NSCLC中占到1%-2%，和*ROS1*、*c-MET*等其他驱动基因变异一样，属于相对少见而并不罕见。由于我国NSCLC患者的基数庞大，*RET*融合阳性患者的绝对数量非常大。目前对*RET*基因融合检测的推荐已经在很多国内外指南和共识中有所体现。2020年中国抗癌协会临床肿瘤学协作中心(Chinese Society of Clinical Oncology, CSCO)NSCLC诊疗指南对不可手术和晚期NSCLC患者进行*RET*基因检测(II级推荐)。分子检测样本以组织为主，如果组织不够或无法获取，可以通过血液循环肿瘤DNA(circulating tumor DNA, ctDNA)检测。研究表明ctDNA特异性可以接近100%，但是敏感性略差，存在假阴性。而ctDNA检测阴性的患者强调要尽可能再次进行组织检测。基因融合的检测主要基于3个层面(蛋白、DNA和RNA); 包括4种方法即免疫组织化学(immunohistochemistry, IHC)、荧光原位杂交(fluorescence *in situ* hybridization, FISH)、逆转录聚合酶链反应(reverse transcriptase polymerase chain reaction, RT-PCR)及NGS; 现在FISH是融合检测的金标准。因为融合在肺癌中发生率比较低，FISH用于初筛的性价比不高，而作为第二平台的验证非常重要。IHC在*ALK*等融合基因中已经广泛应用，但是RET抗体的敏感性和特异性均很低，所以IHC作为初筛还有待于进一步的临床研究验证，评判标准和与其他平台的一致性还需要更多的临床实践证实。目前主要推荐RT-PCR及NGS方法检测，NGS包括DNA NGS(可同时检测多个突变，但内含子区域覆盖率低，降低其检测敏感性)及RNA NGS(其优势为不存在内含子覆盖率问题，同时可获得融合伴侣信息，直接检测融合表达)。大多数的*RET*融合能通过NGS检测到，尤其是，RNA NGS检测敏感性更高^[31]^。美国国立综合癌症网络(National Comprehensive Cancer Network, NCCN)肺癌指南推荐诊断时采用广谱NGS同时检测*RET*融合、*EGFR*、*ALK*、*ROS1*、*BRAF* V600E、*NTRK*及*MET* ex14变异。使用NGS技术检测我国肺癌患者，*RET*融合阳性的发生率为1.4%^[32]^。

## 3 RET融合型NSCLC患者的临床病理特征

*RET*融合型NSCLC患者的具有独特的临床病理特征，发病年轻(≤60岁)，不吸烟，与性别相关性不大; 病理学发现腺癌多见，肿瘤细胞中印戒细胞比例≥10%，体积更小(≤3 cm)，淋巴结转移更早，低分化多见^[33]^，病理多为乳突状或鳞屑状形态，与放射性暴露的相关性有争议^[34-36]^，同时伴有其他致癌性驱动基因改变的可能性小。我国学者^[32]^的一项回顾性研究收集6, 125例应用NGS检测的中国肺癌患者，分析*RET*融合患者的临床特征，男性占42.9%，女性占56%。中位年龄58岁(35岁-81岁); 组织类型：腺癌为72.8%，鳞癌为1.2%，腺鳞混合癌为3.6%，其他类型为21.4%。*RET*融合伴侣类型：*KIF5B*为54%、*CCDC6*为17%、*EML4*和*NCOA4*为3%，其他为26%。

## 4 RET融合型NSCLC治疗进展

2012年首次在NSCLC中发现*RET*融合靶点，2016年多靶点抑制剂卡博替尼(Cabozantinib)、凡德他尼(Vandetanib)治疗*RET*融合阳性NSCLC的III期临床研究相继发表，2017年特异性RET抑制剂开始进行临床试验，BLU-667和LOXO-292在*RET*融合阳性NSCLC中展现出强大活性，2020年5月LOXO-292被美国食品药品监督管理局(Food and Drug Administration, FDA)批准上市，随后，2020年9月4日普拉替尼(BLU-667)获FDA批准上市。

特异性RET抑制剂出现以前，*RET*融合NSCLC从现有治疗手段中显著获益有限。含培美曲塞化疗方案的客观缓解率(objective response rates, ORR)45%和中位无进展生存(median progression-free survival, mPFS)9个月，与*ALK*及*ROS1*重排型肺癌一致^[37]^。2020年中国多中心回顾性研究^[38]^，2011年-2018年，10家医院共入组62例*RET*融合NSCLC患者，50例为IIIb期/IV期，40例接受了一线化疗，28例接受了二线化疗，评估化疗方案的疗效。结果显示：一线化疗PFS为5.2个月-9.2个月; 二线化疗PFS为2.8个月-4.9个月。

*RET*融合NSCLC同样未能从免疫治疗中获得显著疗效，*RET*融合肺癌患者中PD-L1表达情况报道差异极大(0%-70%)^[39-41]^。真实世界回顾性研究^[42]^，21个中心入组107例晚期NSCLC患者，其中9例*RET*融合，接受免疫检查点抑制剂(immune checkpoint inhibitors, ICIs)治疗ORR为37.5%，PFS为7.6个月。另一项回顾性研究^[43]^，纳入10个国家24个中心551例接受ICI单药治疗的具有至少一个致癌性驱动突变的晚期NSCLC患者，其中RET融合患者16例，ORR为6.3%，PFS为2.1个月。

临床尝试多激酶抑制剂(multikinase inhibitors, MKIs)[凡德他尼(Vandetanib)^[44]^、舒尼替尼(Sunitinib)^[44]^、索拉非尼(Sorafenib)^[44]^、卡博替尼(Cabozantinib)^[44]^、仑伐替尼(Lenvatinib)^[45]^、阿来替尼(Alectinib)^[46]^、多维替尼布(Dovitinib)^[47]^、普纳替尼(Ponatinib)^[48]^及阿帕替尼(Apatinib)^[49]^]治疗*RET*变异NSCLC，然而这些TKIs仅有中等程度的缓解率，缓解持续时间有限，毒性也较为显著。如卡博替尼及凡德它尼治疗*RET*融合型NSCLC，卡博替尼的ORR和中位PFS分别为28%及5.5个月^[50]^;而凡德它尼分别为18%及4.5个月^[51]^;均明显低于ALK(83%及25.7个月)^[52]^，*ROS1*(mPFS：9.1个月-19.3个月)基因融合和*EGFR*突变(80%及18.9个月)^[53]^NSCLC患者靶向治疗的数据，与细胞毒化疗取得的数据相似; 而且MKIs 3级/4级毒性发生率高，70%以上的患者需要减量。分析MKIs疗效低下且高毒性的研究发现^[14]^，从生化和细胞分析来看，多靶点RET抑制剂对全长、野生型RET具有不同的活性，其效价也不同; 另外，生化半抑制浓度(biochemical half-maximal inhibitory concentration, IC_50_)在 < 5 nmol/L-100 nmol/L范围内，如卡博替尼的IC_50_对于血管内皮生长因子受体2(vascular endothelial growth factor receptor 2, VEGFR2)和RET分别为0.035 nmol/L及5.2 nmol/L，卡博替尼对于RET的标准剂量下，由于VEGFR2抑制引起的不良反应如高血压，蛋白尿，手足综合征等明显增加。因此，MKIs靶向RET及非-RET包括VEGFR2等多靶点的同时，导致的不良反应限制其长期应用及高选择性RET的充足剂量。

由广东省人民医院杨衿记^[41]^教授团队牵头，联合全国13家中心，共同完成一项对携带*RET*融合NSCLC人群、中国最大的多中心回顾性研究，共计129例患者，大多数患者的肿瘤原发灶-淋巴结-转移(tumor-node-metastasis, TNM)分期为III期-IV期(*n*=110, 85.3%)，45例晚期患者的总生存期(overall survival, OS)为20.3个月; 接受3种不同治疗的患者，其中PFS无明显差异(中位PFS：MKIs：3.8个月; ICIs：2.5个月; 化疗：3.5个月)。综上，临床迫切需要高效高选择性且低毒性的针对*RET*基因的抑制剂。

随后，特异性RET抑制剂被研发出来，为晚期*RET*基因重排的NSCLC患者带来了希望。I期/II期LIBRETTO-001研究^[54]^中，评估selpercatinib对RET驱动型NSCLC患者的疗效，结果显示：105例既往接受过含铂双药化疗晚期患者的ORR为64%，39例未经含铂双药化疗初治患者组的ORR为85%，10例颅内转移患者的ORR为91%;中位PFS为18.4个月，1年PFS率为68%;最常见的3级或4级不良事件是高血压、转氨酶升高、低钠血症和淋巴细胞减少; 30%患者需要减少剂量，而2%患者由于毒性而停药。因为Selpercatinib在初治、经治及合并脑转移的*RET*融合NSCLC患者中均显示了具有临床意义的缓解和持续抗肿瘤活性，且毒性可耐受，因此，2020年5月8日，FDA加速批准selpercatinib用于*RET*融合基因阳性的转移性NSCLC患者。

Pralsetinib(普拉替尼)是另一种选择性RET-TKI，在*RET*融合型NSCLC中的研究结果令人印象深刻。ARROW试验是一项I期/II期研究^[55]^，评估了Pralsetinib在*RET*融合肿瘤患者中的作用。结果显示，80例经治患者(占49%，其中32%接受过3线及以上化疗)的ORR为61%，26例初治疗患者的ORR为73%。无论*RET*基因融合类型、既往是否接受免疫检查点抑制剂治疗，患者均可观察到治疗反应。缓解持续时间(duration of response, DoR)未达到，6个月DoR率为83%。最常见的不良事件(3级及以上)包括中性粒细胞减少、高血压和贫血。2020年9月，FDA加速批准Pralsetinib治疗转移性*RET*融合阳性NSCLC患者。2021年3月获得国家药品监督管理局(National Medical Products Administration, NMPA)批准既往接受过含铂化疗的*RET*基因融合阳性的局部晚期或转移性NSCLC成人患者。这些RET抑制剂已经改变了*RET*融合型NSCLC的治疗前景，并取代了化疗方案，成为*RET*变异NSCLC患者的一线治疗方法。

## 5 RET抑制剂耐药后研究进展

临床前研究和早期临床报告^[56, 57]^报道了多重耐药突变。MKIs如卡博替尼可诱导一种门控(gatekeeper)突变，即V804L耐药突变，而凡德它尼和选择性RET抑制剂Selpercatinib可诱导溶解前突变(G810A/S)。一项临床研究^[58]^发现，一例*KIF5B-RET*融合型NSCLC患者对Selpercatinib有显著的初始反应后，ctDNA分析提示*RET*溶解前突变包括*RET* G810R、G810S和G810C出现在临床耐药之前。尸检报道了在多个疾病部位含有G810S、G810R和G810C突变，表明G810残基的进化导致了一种共同的耐药机制。在Selpercatinib的I期/II期研究^[54]^中发现，*CCDC6-RET*融合型NSCLC患者的肿瘤组织及RET另一融合型NSCLC及*RET*突变的MTC患者的血浆中发现了另一耐药机制即*RET* G810的获得性突变。

TPX-0046^[55]^是一种选择性的下一代RET/SRC抑制剂，与Selpercatinib和Prasetinib在结构上存在差异，酶联分析发现，TPX-0046可以拮抗野生型和各种突变的RET以及SRC，但对VEGFR2无效。TPX-0046在基因工程鼠Ba/F3 KIF5B-RET、TT和LC2/ad细胞中有效抑制RET磷酸化和细胞增殖，IC_50_约为1 nmol/L。

TPX-0046在Ba/F3细胞增殖中可以拮抗SFM-G810R，且IC_50_为17 nmol/L，而BLU-667和LOXO-292的IC_50_ > 500 nmol/L。TPX-0046在体内多种RET驱动的癌细胞和患者来源的异种移植肿瘤模型中显示出显著的抗肿瘤疗效。目前尚需开展进一步的临床研究。

总之，*RET*融合型NSCLC发病率低，选择性靶向治疗给患者带来了希望，通过NGS基因组测序确定患者亚型至关重要。近年来针对*RET*融合型NSCLC的分子靶向治疗取得了重大成功，但由于不可避免地会发生耐药，因此患者的受益一直受限。为提高疗效，克服耐药，减少毒性，还需要进一步的研究探索耐药机制并发现更多有效的靶向治疗药物。
